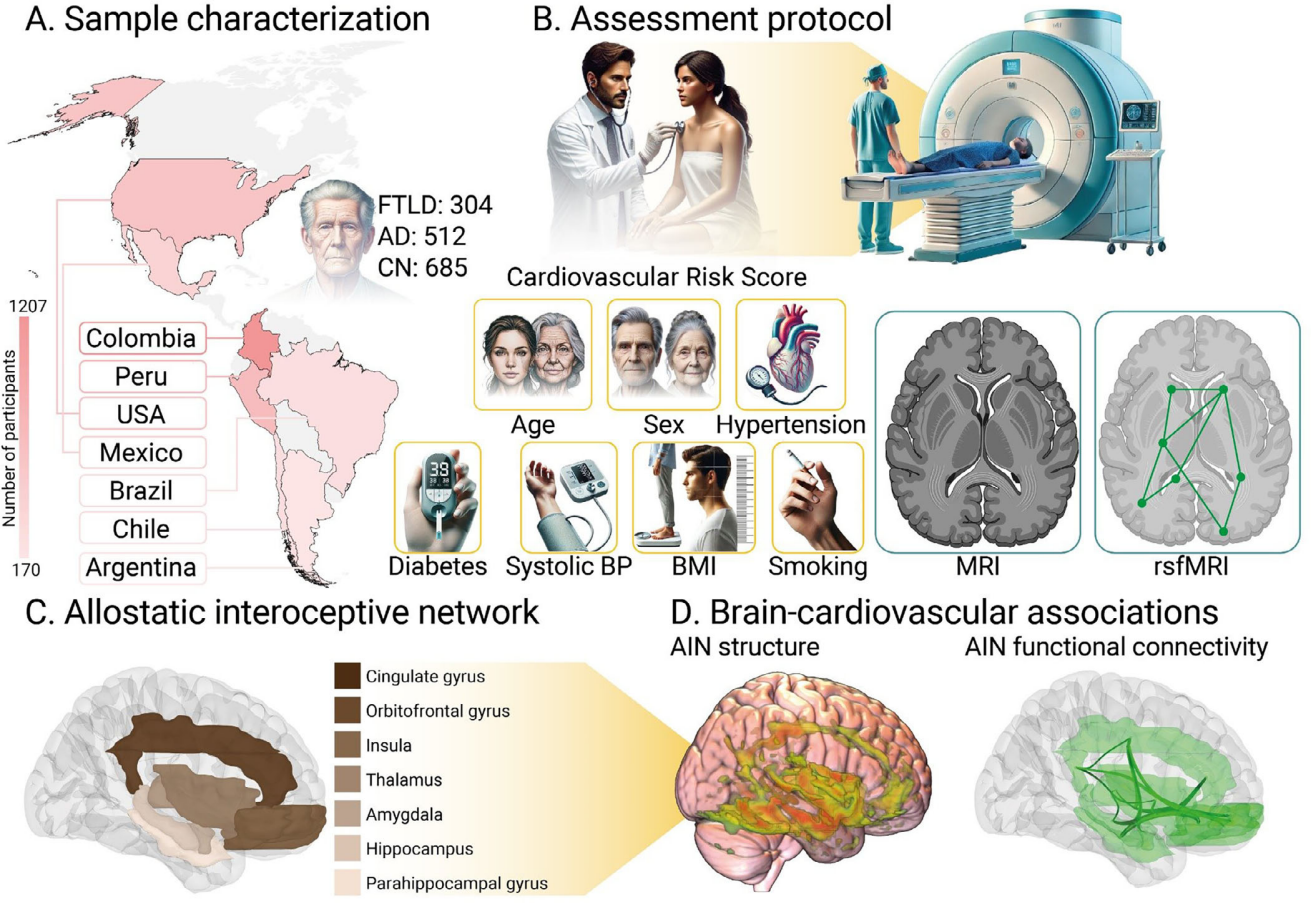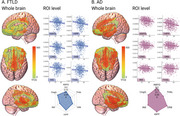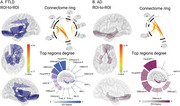# Cardiovascular risk factors and the allostatic interoceptive network in dementia

**DOI:** 10.1002/alz70862_109908

**Published:** 2025-12-23

**Authors:** Jessica L Hazelton, Joaquín Migeot, Raul Gonzalez‐Gomez, Florencia Altschuler, Claudia Duran‐Aniotz, Olivia Wen, Dante Sebastián Galván Rial, Pablo Barttfeld, Vicente Medel, Cecilia Gonzalez Campo, Ana Maria Castro Laguardia, Hernan Hernandez, Carolina Gonzalez‐Silva, Olga Castaner, Kun Hu, Peng Li, María Isabel Behrens, Martin Alejandro Bruno, Juan Cardona, Nilton Custodio, Hernando Santamaria‐Garcia, Adolfo M Garcia, Maria Eugenia Godoy, José Alberto Ávila Funes, Marcelo Adrian Maito, Diana L Matallana, Bruce L. Miller, Francisco Lopera, Maira Okada de Oliveira, Stefanie Pina‐Escudero, Katherine L. Possin, Elisa de Paula, França Resende, Pablo A Reyes, Andrea Slachevsky, Ana Luisa Sosa, Leonel Tadao Takada, Jennifer S. Yokoyama, Agustin Ibanez

**Affiliations:** ^1^ Latin American Brain Health Institute (BrainLat), Universidad Adolfo Ibañez, Santiago Chile; ^2^ Cognitive Neuroscience Center (CNC), Universidad de San Andrés, Buenos Aires, Buenos Aires Argentina; ^3^ The University of Sydney, Brain and Mind Centre, Sydney, NSW Australia; ^4^ Latin American Brain Health Institute (BrainLat), Universidad Adolfo Ibáñez, Santiago, Región Metropolitana de Santiago Chile; ^5^ Global Brain Health Institute (GBHI), University of California San Francisco (UCSF); & Trinity College Dublin, Dublin, Leinster Ireland; ^6^ Instituto de Investigaciones Psicológicas (IIPsi, CONICET‐UNC), Facultad de Psicología, Universidad Nacional de Córdoba, Córdoba, Córdoba Argentina; ^7^ CONICET, Buenos Aires Argentina; ^8^ Cardiovascular risk and Nutrition Research Group. Hospital del Mar Research Institute, Spain; CIBER Epidemiology and Public Health (CIBERESP), ISCIII, Spain, Barcelona, Barcelona Spain; ^9^ Harvard Medical School, Boston, MA USA; ^10^ Massachusetts General Hospital, Boston, MA USA; ^11^ Facultad de Medicina, Universidad de Chile, Santiago, Región Metropolitana de Santiago Chile; ^12^ Departamento de Neurología y Neurocirugía, Hospital Clínico, Universidad de Chile., Santiago, Región Metropolitana de Santiago Chile; ^13^ Centro de Investigación Clínica Avanzada (CICA), Universidad de Chile, Santiago, Región Metropolitana de Santiago Chile; ^14^ Clínica Alemana‐Universidad del Desarrollo, Santiago, Región Metropolitana de Santiago Chile; ^15^ Instituto de Ciencias Biomédicas (ICBM) Facultad de Ciencias Médicas, Universidad Catoóica de Cuyo, San Juan, San Juan Argentina; ^16^ Universidad del Valle, Cali, Valle del Cauca Colombia; ^17^ Unidad de Investigación de Deterioro Cognitivo y Prevención de Demencia, Instituto Peruano de Neurociencias, Lima, Lima Peru; ^18^ Pontificia Universidad Javeriana, Bogotá Colombia; ^19^ Center for brain and memory intellectus, Bogotá Colombia; ^20^ Global Brain Health Institute (GBHI), University of California San Francisco (UCSF); & Trinity College Dublin, San Francisco, CA USA; ^21^ Global Brain Health Institute (GBHI), University of California San Francisco (UCSF); & Trinity College Dublin, Dublin Ireland; ^22^ Universidad de Santiago de Chile, Santiago, Santiago Chile; ^23^ Instituto Nacional de Ciencias Médicas y Nutrición Salvador Zubirán, Mexico City, DF Mexico; ^24^ Pontificia Universidad Javeriana, Bogota, Cundinamarca Colombia; ^25^ Hospital Universitario San Ignacio, Bogotá, Bogotá Colombia; ^26^ Department of Neurology, Memory and Aging Center, University of California San Francisco, San Francisco, CA USA; ^27^ Grupo de Neurociencias de Antioquia, Facultad de Medicina, Universidad de Antioquia, Medellín, Antioquia Colombia; ^28^ Cognitive Neurology and Behavioral Unit (GNCC), University of Sao Paulo, Sao Paulo, Sao Paulo Brazil; ^29^ Memory and Aging Center, University of California San Francisco, San Francisco, CA USA; ^30^ Universidade Federal de Minas Gerais, Belo Horizonte, Minas Gerais Brazil; ^31^ Instituto de Envejecimiento, Facultad de Medicina, Pontificia Universidad Javeriana, Bogotá, Bogotá Colombia; ^32^ Universidad de Chile, Santiago, Región Metropolitana de Santiago Chile; ^33^ Neurology Service, Department of Medicine, Clínica Alemana, Universidad del Desarrollo, Santiago, Región Metropolitana de Santiago Chile; ^34^ Neurology Department, Hospital del Salvador, University of Chile, Santiago, Región Metropolitana de Santiago Chile; ^35^ Geroscience Center for Brain Health and Metabolism (GERO), Santiago, Región Metropolitana de Santiago Chile; ^36^ Instituto Nacional de Neurología y Neurocirugía, Mexico City, DF Mexico; ^37^ Hospital das Clínicas, University of Sao Paulo Medical School, São Paulo, São Paulo Brazil; ^38^ Memory and Aging Center, Department of Neurology, Weill Institute for Neurosciences, University of California, San Francisco, San Francisco, CA USA; ^39^ Department of Radiology and Biomedical Imaging, University of California, San Francisco, San Francisco, CA USA

## Abstract

**Background:**

Cardiovascular risk factors, such diabetes, hypertension, blood pressure, obesity, and smoking, are linked with allostatic‐interoception – the continuous monitoring of internal bodily states in anticipation of environmental demands. These risk factors are associated with dementia risk. How these factors affect brain networks vulnerable to neurodegeneration and involved in allostatic‐interoception, such as the Allostatic‐Interoceptive Network (AIN), is unknown. We investigated the relationship between cardiovascular risk and AIN structure and function in frontotemporal lobar degeneration (FTLD) and Alzheimer’s disease (AD).

**Method:**

We recruited 1501 participants (304 with FTLD, 512 with AD, and 685 healthy controls) from the Multi‐Partner Consortium to Expand Dementia Research in Latin America (ReDLat)(Figure 1). A cardiovascular risk score was calculated based on: age, sex, diabetes, hypertension, systolic blood pressure, body mass index, and smoking status. Cardiovascular risk was associated with gray matter integrity and functional connectivity in age‐ and sex‐matched patient‐control groups focusing on predefined regions of interest within the AIN.

**Result:**

Higher cardiovascular risk was associated with reduced structural integrity and functional connectivity within the AIN in both FTLD and AD. In FTLD patients, extensive structural (Figure 2) and functional connectivity disruptions (Figure 3) were observed throughout the AIN. In AD patients, structural reductions in the AIN were prominent (Figure 2), with functional connectivity restricted to the hippocampus, parahippocampal gyrus, and orbitofrontal regions (Figure 3).

**Conclusion:**

Cardiovascular risk factors appear to adversely impact the AIN structure and function, with disease‐specific patterns of vulnerability. Our results underscore the importance of integrating cardiovascular health into models of neurodegenerative disease and managing cardiovascular health to support brain integrity in dementia. Future work is needed to uncover longitudinal effects of cardiovascular risk in dementia and to determine if cardiovascular risk factors exacerbate neurodegenerative processes.